# Delay in Seeking Institutional Delivery Services and Associated Factors Among Immediate Postpartum Mothers in Public Health Facilities in Gondar Town, Northwest Ethiopia, 2022

**DOI:** 10.1089/whr.2024.0014

**Published:** 2024-05-06

**Authors:** Tazeb Alemu Anteneh, Zerfu Mulaw Endale, Getie Mihret Aragaw, Hana Yohanes Mamo, Betelhem Ketema, Temesgen Gashaw, Rahel Misaw, Tiringo Molla Tiruye

**Affiliations:** ^1^ Department of Clinical Midwifery, School of Midwifery, College of Medicine and Health Sciences, University of Gondar, Gondar, Ethiopia.; ^2^ Department of General Midwifery, School of Midwifery, College of Medicine and Health Sciences, University of Gondar, Gondar, Ethiopia.

**Keywords:** delay, Ethiopia, seeking institutional delivery services

## Abstract

**Background:**

Maternal and neonatal mortality is a global problem that is highly prevalent in low- and middle-income countries, including Ethiopia. Maternal delay in seeking institutional delivery services utilization plays a significant role in determining maternal and neonatal health outcomes. Although studies have been conducted on institutional delivery service utilization in Ethiopia, little is known about factors for delays in seeking care for institutional delivery services.

**Objective:**

This study aimed to assess the delay in seeking institutional delivery services and associated factors among immediate postpartum mothers in public health facilities in Gondar, northwest Ethiopia.

**Methods:**

A facility-based cross-sectional study was conducted from July 15 to September 10, 2022. A total of 391 participants were selected using systematic random sampling. Data were collected through face-to-face interviews using structured, pretested, and interviewer-administered questionnaires. Data were entered into EpiData version 4.6, and the analysis was conducted using Statistical Package for Social Science version 26. The multivariable logistic regression model was fitted and the level of significance was set at *p* ≤ 0.05.

**Result:**

The prevalence of delay in seeking institutional delivery was 49.10% (95% confidence interval [CI]: 44.13, 54.08). Rural residence (adjusted odds ratio [AOR] = 2.51; 95% CI: 1.43–4.41), no antenatal care visits (AOR: 2.87; 95% CI: 1.34–6.13), unplanned pregnancy (AOR: 2.98; 95% CI: 1.78–5.01), poor decision-making autonomy in maternity care services (AOR: 1.98; 95% CI: 1.15–3.40), and poor birth preparedness plan (AOR: 4.88; 95% CI: 2.79–8.53) were significantly associated with delays in seeking institutional delivery.

**Conclusion:**

Delays in seeking institutional delivery services were high. It is better to promote women’s decision-making power in their own health care. In addition, it is better to arrange programs that will improve maternal and child health service utilization.

## Introduction

Globally, an estimated 287,000 maternal deaths occurred in 2020, yielding an overall maternal mortality ratio (MMR) of 223 maternal deaths per 100,000 live births.^1^ Almost 99% of these maternal deaths occur in low- and middle-income countries, whereas sub-Saharan Africa accounts for ∼70% of global maternal deaths, with a rate of 545 per 100,000 live births.^1^ Similarly, in Ethiopia, 401 women die per 100,000 live births from pregnancy-related complications, which is significantly higher than the United Nations’ goal of reducing the global MMR to less than 70 per 100,000 live births.^2^ Maternal delay factors, together with other medical factors, play a significant role in determining maternity outcomes; a delay in seeking institutional delivery contributes to 36.1% of maternal deaths in Ethiopia.^3^

Although most maternal and neonatal mortalities are preventable by early recognition and treatment of complications, various barriers affect women’s access to emergency obstetric care services. These barriers are interdependent and occur at different levels, in decision-making to obtain services, on the way to health facilities, or at the facilities.^4,5^ Evidence shows that husbands’ educational status, monthly family income, women’s residence, and educational status contribute to delays in seeking institutional delivery services.^6–8^

Currently, reducing maternal and neonatal mortality is a global priority. One of the sustainable development goals is to reduce the global MMR to less than 70 maternal deaths per 100, 000 live births and the neonatal mortality rate to less than 12 deaths per 1,000 live births by 2030.^9^ Similarly, the Ethiopian Ministry of Health planned to reduce the MMR from 401 to 279 maternal deaths per 100,000 live births by 2024/25.^2^ Different initiatives were implemented to reduce delay-related maternal deaths, such as the establishment of maternity waiting homes in health facilities,^10^ arranging free transportation and maternity care services, and introducing health extension programs.^11^ Despite all these efforts, maternal deaths and morbidities resulting from delays in seeking institutional delivery services remain unacceptably high.^3^ Although several studies have been conducted on institutional delivery service utilization in Ethiopia, little is known about delays in seeking care for institutional delivery services.

Therefore, this study aimed to assess the delay in seeking institutional delivery services and associated factors among immediate postpartum mothers in public health facilities in Gondar, northwest Ethiopia.

## Methods

### Study design, period, and setting

A facility-based cross-sectional study was conducted from July 15 to September 10, 2022, in public health facilities in Gondar, northwest Ethiopia. The administrative town, Gondar, is located ∼760 km northwest of Addis Ababa (the capital city of Ethiopia) and 174 km from Bahir Dar (the capital city of the Amhara regional state). According to Ethiopia’s population projections for all regions at the woreda level from 2014 to 2017, the total population of the town is estimated to be 306,246. Among them, 149,970 were males and 156,276 were females. The town has one comprehensive specialized hospital, one private general hospital, one private primary hospital, and eight health centers that provide maternity and child health services.

### Study population

All immediate postpartum mothers in public health facilities in Gondar town, northwest Ethiopia, who were available during the data collection period, were included.

### Sample size determination and sampling procedure

The sample size was determined using a single population proportion formula considering the following assumptions: The proportion of delay in seeking institutional delivery was 36.3% from a facility-based cross-sectional study conducted in South Gondar zone health facilities.^8^ Ninety-five percent level of confidence, and 5% margin of error were used.

$$n=\frac{\;{\left(Z\alpha /2\right)}^{2}p(1-p)}{d^{2}}\;=\;(1.96)2\text{ * }0.363(1-0.363)/(0.05)2\;$$

*n* = 355

where *n* = required sample size, *z* = standard normal distribution curve value for the 95% confidence level = 1.96, α = level of significance, *p* = proportion of delay in seeking institutional delivery, and *d* = margin of error. Considering a 10% nonresponse rate, the final sample size was 391. Gondar town has eight health centers and one comprehensive specialized hospital. The study participants were selected proportionally using a systematic random sampling technique with an interval size (*k*) of 6: 2050/391 = 5.2 ≈ 6.

## Measurements

Delay in seeking institutional delivery: The time interval between recognition of the labor and deciding to seek institutional delivery service ≥1 hour is considered a delay.^12^

Birth preparedness and complication readiness plan: Birth preparedness and complication readiness have five intervention components: identifying the place of delivery, arranging potential blood donors and skilled birth attendants, arranging means of transport during an emergency, and saving money that can be used during an emergency. Women who practiced at least three of the five components (60%) were considered to be prepared for birth and their complications.^13^

Women’s decision-making autonomy in maternal health care service: A woman is said to be autonomous when she can decide independently or jointly with her partner to attend a maternal health service. If a woman decided independently or jointly, the response was coded as 1, whereas a partner decided alone was coded as 0. Therefore, women who independently or jointly decided with their husbands to answer ≥50% of the questions were categorized as having decision-making autonomy.^14^

Media exposure: Those who responded at least once a week to at least one of the media types (television, radio, or magazine) were considered regularly exposed.^15^

## Data Collection Tools, Procedures, and Quality Control

The data collection tool was developed by reviewing the relevant literature. A structured, interviewer-administered questionnaire was used to collect data through face-to-face interviews. Initially, the questionnaire was prepared in English, translated into the local (Amharic) language, and back translated to English to ensure consistency. The questionnaire comprised questions on sociodemographic characteristics and reproductive and maternity health care characteristics. Three diplomas and one BSc midwife were recruited for the data collection and supervision, respectively. A pretest was performed on 5%^16^ of the sample at the Dembia primary hospital. One-day training was provided for data collectors and supervisors about the aim of the study, contents of the tool, and sampling technique to ensure language clarity and to provide information on interview techniques and on how to keep the information. During the data collection period, the supervisor and investigators checked the questionnaires daily.

## Data Processing and Analysis

The coded data were entered into EpiData version 4.6 and then exported to Statistical Package for Social Science version 26 analysis software for further cleaning and analysis. Descriptive statistics were used to analyze the characteristics of the study participants. A binary logistic regression model was fitted to identify factors associated with delays in seeking institutional delivery. Variables with a *p*-value of ≤0.25 in the bivariable analysis were entered into a multivariable logistic regression analysis to identify independent factors associated with the delay in seeking institutional delivery services. To conduct a multivariable logistic regression analysis, variables were selected using the backward likelihood ratio approach, and model fitness was checked using the Hosmer–Lemeshow test. A *p*-value of ≤0.05, with a 95% confidence interval (CI) for the adjusted odds ratio, was employed to declare a significant association.

## Ethical Considerations

The study was conducted in accordance with the Ethiopian Health Research Ethics Guidelines and Declaration of Helsinki. Ethical approval was obtained from the School of Midwifery on behalf of the Institutional Review Board (IRB) of the University of Gondar. A formal letter of organizational approval was obtained from health facilities. Written informed consent was obtained from each participant before data collection. Finally, the data were coded and electronic data were stored in password-protected computers or files to maintain participant confidentiality.

## Result

### Sociodemographic characteristics

A total of 391 mothers were included in the study, with a response rate of 100%. The median age of the participants was 28 years with an interquartile range of 24–31 years and 74.9% of the respondents were within the age-group of 21 to 34 years. More than two-thirds (70.3%) of mothers were urban residents. In addition, most (89.3%) of the study participants were married and 95% of the study participants had media exposure (Table 1).

**Table 1.** Sociodemographic Characteristics of Study Participants in Gondar Town Public Health Facilities, Northwest Ethiopia, 2022 (*n* = 391)

Characteristics	Category	Frequency	Percentage
Age-group of mothers in year	≤20	35	9.0
	21–34	293	74.9
	≥35	63	16.1
Residence	Urban	275	70.3
	Rural	116	29.7
Religion	Orthodox	249	63.7
	Muslim	92	23.5
	Protestant	41	10.5
	Others^a^	9	2.3
Educational status of the mother	Unable to read and write	56	14.3
	Able to read and write	50	12.8
	Primary^1–8^	67	17.1
	Secondary^9–12^	90	23.0
	college and above	128	32.7
Occupational status of the mother	Housewives	203	51.9
	Government employee	64	16.4
	Merchant	41	10.5
	Farmer	47	12.0
	Farmer	36	9.2
Current marital status	Married	349	89.3
	Unmarried	42	10.7
Husband’s occupational status (*n* = 349)	Government employee	90	25.9
	Merchant	90	25.9
	Self-employed	87	25.0
	Farmer	82	23.3
Husband’s educational status (*n* = 349)	Unable to read and write	44	12.5
	Able to read and write	42	11.9
	Primary^1–8^	33	10.5
	Secondary^9–12^	69	19.9
	College and above	158	45.2
Family monthly income (in birr)	≤1,000	32	8.2
	1,001–1,999	38	9.7
	≥2,000	321	82.1
Media exposure	No	19	5.0
	Yes	372	95.0

^a^ Catholic and Seventh-day Adventist.

### Reproductive history and maternity health service-related factors

Nearly three-fourths (72.6%) of the study participants had a planned pregnancy for the most recent pregnancy. Two-thirds (67.3%) of study participants were multiparous. Similarly, three-quarters (74.7%) of participants had decision-making autonomy regarding maternity care services. Slightly less than two-thirds of mothers (63.9%) had poor birth preparedness plans (Table 2).

**Table 2.** Reproductive and Maternal Health Service-Related Characteristics of Study Participants in Gondar Town Public Health Facilities, Northwest Ethiopia, 2022 (*n* = 391)

Characteristics	Category	Frequency	Percentage
Type of the most recent pregnancy	Planned	284	72.6
	Unplanned	107	27.4
Parity	Premipara	127	32.7
	Multipara	264	67.3
ANC utilization	No	60	15.3
	Yes	331	84.7
Number of ANC visits (*n* = 331)	≤3	130	39.3
	≥4	201	60.7
Previous pregnancy outcome (*n* = 264)	Live birth	231	87.8
	Stillbirth	33	12.2
Mode of previous delivery (*n* = 264)	SVD	175	66.2
	Instrumental assisted	39	14.8
	C/S	50	19.0
Place of previous delivery (*n* = 264)	At home	39	14.8
	On transportation	12	4.5
	Health facility	213	80.7
Time of onset of labor during the most recent pregnancy	Day	264	67.5
	Night	127	32.5
Travel time to reach the nearby health facility	<1 hour	348	89.0
	≥1 hour	43	11.0
Access of transportation	Yes	309	79.0
	No	82	21.0
Birth preparedness and complication readiness plan	No	250	63.9
	Yes	141	36.1
Women’s decision-making autonomy of maternity care services	No	99	25.3
	Yes	291	74.7

ANC, antenatal care; C/S, cesarean section; SVD, spontaneous vertex delivery.

### Delay in seeking institutional delivery

The prevalence of delays in seeking institutional delivery was 49.10% (95% CI: 44.13, 54.08).

### Factors associated with delay in seeking institutional delivery

Bivariable and multivariable logistic regression analyses were performed to identify the factors associated with delays in seeking institutional delivery.

In the multivariable logistic regression analysis, rural residence, no birth preparedness and complication readiness plan, unplanned pregnancy, no antenatal care (ANC) visit, and poor women’s decision-making autonomy regarding maternal care services were significantly associated with delays in seeking institutional delivery.

In this study, the odds of delay in seeking institutional delivery were 2.51 times higher among rural residents than urban resident mothers (adjusted odds ratio [AOR] = 2.51; 95% CI: 1.43–4.41). Similarly, unplanned pregnancy increases the odds of delay in seeking institutional delivery by 2.98 times compared with planned pregnancy (AOR: 2.98; 95% CI: 1.78–5.01). The odds of delay in seeking institutional delivery were 2.87 times higher among participants who had no ANC visit than among those who did not (AOR: 2.87; 95% CI: 1.34–6.13). In addition, the odds of delay in seeking institutional delivery were 1.98 times more likely among women who had poor decision-making autonomy on maternity care services (AOR: 1.98; 95% CI: 1.15–3.40). Finally, the odds of delay in seeking institutional delivery were 4.88 times higher among mothers who had poor birth preparedness and complication readiness plans than among their counterparts (AOR: 4.88; 95% CI: 2.79–8.53) (Table 3).

**Table 3.** Factors Associated with Delay in Seeking Institutional Delivery in Gondar Town Public Health Facilities, Northwest Ethiopia, 2022

Variables	Category	Delay in seeking institutional delivery		COR (95% CI)	AOR (95% CI)
		Yes	No		
Residence	Urban	113	162	1	1
	Rural	79	37	3.06 (1.94, 4.84)	2.51 (1.43, 4.41)^*^
Educational status of women	Unable to read and write	43	13	5.16 (2.53, 10.55)	2.82 (0.86, 7.34)
	Able to read and write	25	25	1.56 (0.81, 3.01)	1.08 (0.47, 2.44)
	Primary education	31	36	1.34 (0.74, 2.44)	0.84 (0.42, 1.68)
	Secondary Education	43	47	1.43 (0.83, 2.46)	1.33 (0.72, 2.47)
	College and above	50	78	1	1
Status of pregnancy	Planned	122	162	1	1
	Unplanned	70	37	2.50 (1.57, 3.97)	2.98 (1.78, 5.01)^*^
Mode of previous delivery (*n* = 264)	SVD	96	79	1.82 (0.96, 3.46)	1.98 (0.93, 2.44)
	Instrumental Assisted	16	23	1.04 (0.45, 2.45)	1.60 (0.58, 4.38)
	C/S	20	30	1	1
Previous place of delivery (*n* = 264)	At home	31	8	4.86 (2.14, 11.08)	1.68 (0.56, 5.05)
	On transportation	7	5	1.56 (0.54, 5.72)	0.63 (0.16, 2.44)
	Health facility	94	118	1	1
ANC service utilization	No	44	16	3.40 (1.84, 6.27)	2.87 (1.34, 6.13)^*^
	Yes	148	183	1	1
Traveling time to reach the nearby health facility	<1 hour	157	191	1	1
	≥1 hour	35	8	5.32 (2.40, 11.81)	1.08 (0.93, 2.40)
Decision-making autonomy or the mother	No	64	35	2.34 (1.46, 3.76)	1.98 (1.15, 3.40)^*^
	Yes	128	164	1	1
Birth preparedness	No	139	111	2.08 (1.36, 3.17)	4.88 (2.79, 8.53)^*^
	Yes	53	88	1	1
Media exposure	No	16	3	5.94 (1.70, 10.73)	1.07 (0.24, 4.65)
	Yes	176	196	1	1

^*^ *p* ≤ 0.005.

ANC, antenatal care; AOR, adjusted odds ratio; CI, confidence interval; COR, crude odds ratio; C/S, cesarean section; SVD, spontaneous vertex delivery.

## Discussion

This facility-based cross-sectional study assessed the delay in seeking institutional delivery and associated factors among the immediate postpartum mothers in Gondar town public health facilities in northwest Ethiopia. Nearly half of the mothers experienced a delay in seeking institutional deliveries. Rural residence, no ANC visit, unplanned pregnancy, poor women’s decision-making autonomy regarding maternity care services, and poor birth preparedness plans were significantly associated with delays in seeking institutional delivery.

The prevalence of delay in seeking institutional delivery was 49.1%, which was in line with a study conducted at the Jimma Medical Center (46.7%). The possible justification for this agreement could be the similarity of some maternal and child health service utilizations. In a study conducted in Jimma, the utilization of antenatal care services was 87.4%, which is similar to the findings of this study (84.7%).^7^

On the other hand, the prevalence of delay in seeking institutional delivery services was higher than in studies conducted in South Gondar zone public hospitals (36.3%)^8^ and Dawuro zone public health facilities (42%).^6^ The possible justification for this variation could be the differences in the sociodemographic and reproductive characteristics of the study participants. In a study conducted in the southern Gondar zone, 20.6% of the participants were homemakers by occupation, whereas 51.9% of the participants in this study were homemakers. Evidence shows that housewives have less decision-making autonomy in health care service utilization and other household activities,^17^ in which women’s involvement in health care-related decisions is an important determinant in seeking institutional delivery services. Similarly, in a study conducted in South Gondar zone public health facilities, 81.1% of the study participants had birth preparedness and complication readiness plans, whereas only 36.1% of the study subjects had birth preparedness plans. Therefore, delays in seeking institutional delivery services have increased.

This study found that ANC visits were significantly associated with delays in seeking institutional deliveries. Having no ANC visit increases the delay in seeking institutional delivery by 2.87 times compared to its counterpart. A possible explanation might be that antenatal care is a significant intervention in contributing women into contact with the health system and helping the mothers and their families obtain information from the care providers on birth preparedness and complication plans that can be an opportunity to promote the benefit of skilled attendance and hospital-based delivery.^18^ In addition, having ANC visits might reflect the woman’s concern for her pregnancy and the need for frequent ANC visits that increase their familiarity with medical personnel, which is more likely to increase their seeking behavior for delivery services.^19^

This study confirmed that women’s residence was significantly associated with delays in seeking institutional delivery services. Thus, the odds of delays in seeking institutional delivery services were 2.51 times higher among rural residents than among urban residents. This finding was supported by a study conducted in Dawuro zone public health facilities in southern Ethiopia and South Gondar zone hospitals. A possible explanation could be the differences in the characteristics of rural residents. They may be less educated and may have poor media exposure and access to information on health care services, poor knowledge of complications during labor and childbirth, and poor physical accessibility and availability of health care services.^20^ Similarly, rural residents lack empowerment regarding early decision-making autonomy in maternal and child health services.^16^ Therefore, they may delay seeking institutional delivery.

Women’s autonomy in decision-making regarding maternal and child health services was significantly associated with delays in seeking institutional delivery services. The odds of delay in seeking institutional delivery were 1.98 times more likely among women who did not have decision-making autonomy compared with their counterparts. This could be attributed to the threat of men’s independent decision-making within the family, which might make it impossible for women to have the opportunity to voice their minds, thereby influencing care seeking and service utilization.^21^ On the other hand, autonomous women would make the right choices and access quality health care at all times, particularly during labor and delivery.^22^

The odds of delay in seeking institutional delivery were 2.98 times higher among mothers who had unplanned pregnancies than among their counterparts. A possible justification might be that unplanned pregnancy could discourage women who are likely to reduce maternity health care-seeking behavior.^23^ In addition, women who are less prepared financially and emotionally to meet the demands of pregnancy and childbearing may face challenges in seeking maternal and child health care services.^24^

Finally, birth preparedness and complication readiness plans were significantly associated with delays in seeking institutional delivery services. The odds of delay in seeking institutional delivery were 4.88 times more likely among women who had a birth preparedness plan compared to their counterparts. This finding is supported by a study conducted in public health facilities in the Gamo zone.^25^ The possible explanation could be that the promotion of facility-based delivery is one component of a birth preparedness plan that provides information for women and their families on how to access the service, when seeking institutional delivery services, and the benefits of timely decisions to receive the care.^26^

## Limitation of the Study

This study may have had social desirability bias. To reduce this bias, participants were informed of the purpose of the study and ways of maintaining confidentiality. Despite this limitation, our findings provide important information on delays in seeking institutional delivery services.

## Conclusion

The prevalence of delays in seeking institutional delivery services was high. Rural residence, no ANC visit, unplanned pregnancy, poor women’s decision-making autonomy regarding maternity care services, and poor birth preparedness plans were factors significantly associated with delays in seeking institutional delivery services. Therefore, stakeholders should target women who do not freely decide on their own health care and need to promote their decision-making power. In addition, it is better to arrange programs that will improve maternal and child health service utilization.

## Data Availability

The datasets for this study are available from the corresponding author and can be submitted for reasonable requests.

## Abbreviations Used

ANC: antenatal care
AOR: adjusted odds ratio
CI: confidence interval
COR: crude odds ratio
C/S: cesarean section
MMR: maternal mortality ratio
SVD: spontaneous vertex delivery
